# Effect of Universal Antibiotic Prophylaxis on Prevalence of Surgical Site Infection After Cesarean Section

**DOI:** 10.3390/jcm14207232

**Published:** 2025-10-14

**Authors:** Anja Čopi Jerman, Janja Zver Skomina, Miha Lučovnik, Samo Jeverica

**Affiliations:** 1 Department of Obstetrics and Gynecology, Izola General Hospital, 6310 Izola, Slovenia; 2 Department of Perinatology, University Medical Centre Ljubljana, 1000 Ljubljana, Slovenia; miha.lucovnik@kclj.si; 3 Department of Cardiology, Izola General Hospital, 6310 Izola, Slovenia; 4 Faculty of Health Sciences, University of Primorska, 6000 Koper, Slovenia

**Keywords:** cesarean section, surgical site infection, hospital acquired infections, antibiotic prophylaxis, epidemiology, Slovenia

## Abstract

**Background/Objectives**: Cesarean section (CS) is among the most common surgical procedures worldwide and is associated with a markedly increased risk of postpartum infection, including surgical site infection (SSI). International guidelines recommend routine prophylaxis for all CSs, but in Slovenia, it has traditionally been reserved for high-risk procedures, with limited SSI surveillance data. The aim of this study was to determine the incidence of SSI within 30 days after CS and to evaluate the impact of universal prophylaxis implemented in a regional secondary care teaching hospital. **Methods**: We conducted a retrospective observational cohort study including all CS performed during 2023 (risk-based-only prophylaxis) and 2024 (universal prophylaxis) at Izola General Hospital, Slovenia. SSI was defined according to ECDC criteria and identified from inpatient and outpatient records up to 30 days postoperatively. Logistic regression was used to assess associations between prophylaxis, clinical variables, and SSI. **Results**: Among 1055 deliveries (208 CS; 99 in 2023, 109 in 2024), the rate of antimicrobial prophylaxis increased from 58.6% to 89.0% (*p* < 0.001). The overall 30-day SSI incidence was 7.2%, with no significant difference between the pre- and post-implementation periods (8.1% vs. 6.4%, *p* = 0.644). Most infections (86.7%) were diagnosed after discharge and were superficial incisional SSI (60%). In multivariable analysis, prophylaxis was independently protective (adjusted OR 0.11; 95% CI 0.02–0.58; *p* = 0.009), while prelabor rupture of membranes (PROM) and higher maternal weight significantly increased SSI risk. **Conclusions**: Antibiotic prophylaxis was independently associated with a reduced risk of SSI following SC; however, the absolute infection rate did not decline significantly and remained moderate after implementation. PROM and higher maternal weight were additional independent risk factors. These findings support universal prophylaxis with optimization for high-risk women and ongoing hospital and national surveillance to improve CS safety.

## 1. Introduction

Cesarean section (CS) is among the most frequently performed surgical interventions worldwide and plays a vital role in perinatal care by preventing maternal and neonatal morbidity and mortality, particularly in complex clinical situations such as obstructed labor or fetal distress [1,2]. Globally, the use of CS has increased substantially, rising from 12% of births in 2000 to an estimated 21% in 2015, with projections indicating it could reach 29% by 2030 [3,4]. Significant regional disparities are evident, with rates varying from below 1% in certain low-income countries to exceeding 50% in others, with substantially higher utilization among women of higher socioeconomic status and in private healthcare settings, many of which are performed with no medical or obstetric indication [5,6]. In Slovenia, 21.9% of births were performed by CS between 2015 and 2019, placing the country among those with relatively low prevalence compared to the European average of 26.0%; however, its use in Slovenia continues to rise, unlike in many European countries where rates have plateaued [7].

Although essential for safe maternal and neonatal outcomes when clinically indicated, CS carries up to five times higher risk of postpartum infections compared to vaginal birth which most commonly comprise surgical site infection (SSI), endometritis and urinary tract infection [8,9,10]. The rate of SSI post-CS in high-income countries ranges from below 1% to over 10% and it ranks high in comparison to other surgical procedures [11,12,13]. Several factors have been shown to contribute to increased risk of SSI after CS among which emergency surgery, prolonged labor, ruptured membranes, low socioeconomic status, limited prenatal care, obesity, surgical technique, and others [14]. Preoperative antimicrobial prophylaxis is one of the most successful interventions for preventing SSI in surgery and is most effective when given before skin incision [15,16]. It has been successfully used in CS since the 1980s, initially for CSs with a higher risk of infection and later, when more data were available, also for lower risk elective cases [14,17,18]. It has also been shown that an additional advantage is obtained if antibiotics are not withheld until the cord clamping is performed with no added neonatal risk [19,20]. Currently, most international guidelines recommend the use of antimicrobial prophylaxis for all cesarean deliveries [21,22,23].

In Slovenia, preoperative antibiotic prophylaxis remains largely limited to CSs at higher risk of SSI, mainly emergency procedures. At the same time, systematic monitoring of SSI following CS is not routinely performed at a national level. Furthermore, where surveillance is performed, post-SC SSI rates are mostly reported for the short period until postpartum hospital discharge and are seldom extended to the full 30-day postoperative period as recommended internationally for accurate SSI assessment [24,25]. As a result, existing data are either unavailable or likely underestimate the true burden of infection, limiting the ability to evaluate maternal outcomes and hindering evidence-based changes in clinical practice [13].

The primary aim of this study was to determine the incidence of SSI within 30 days after cesarean delivery during the two-year period before and after the implementation of universal antibiotic prophylaxis for all cesarean deliveries in a regional secondary care teaching hospital. The secondary aim was to evaluate the effect of prophylaxis on SSI occurrence while accounting for potential confounding factors.

## 2. Materials and Methods

### 2.1. Study Design and Clinical Setting

We conducted a retrospective observational cohort study in the Department of Obstetrics and Gynecology at Izola General Hospital (IGH), a community secondary care regional teaching hospital with 300 acute-care beds serving a population of 170,000. The department includes outpatient clinics and labor and gynecology wards with altogether 50 beds and approximately 550 births per year. Hospital’s CS rate has remained stable over the last decade at around 20% with around 45% of CS being elective.

Medical records (i.e., electronic and paper-based) from all CSs performed in the two-year period from 2023 to 2024 were reviewed. Clinical data previously associated with the incidence of SSI were extracted. Among them were maternal age, number of previous deliveries, maternal weight at delivery, maternal body mass index (BMI), duration of surgery, surgeon, emergency vs. elective operation, type of anesthesia, surgical antimicrobial prophylaxis, prelabor rupture of membranes (PROM) to delivery duration, and maternal smoking during pregnancy. The study period was selected because of a change in practice introduced between the two periods. In 2023, only women at high risk, especially those undergoing emergency CS, received surgical antibiotic prophylaxis, while in 2024, following a local review of international recommendations, prophylaxis was extended to all CS cases. Standard antimicrobial prophylaxis consisted of cefazolin (2 g) administered within 60 min prior to skin incision, with clindamycin (600 mg) used as an alternative in women with history of beta-lactam allergy and was not withheld until cord clamping.

SSIs were defined according to the European Centre for Disease Prevention and Control (ECDC) and Centers for Disease Control and Prevention (CDC) definitions, i.e., occurring within 30 days of surgery and meeting at least one diagnostic condition for each SSI type: (i) superficial incisional SSI (purulent drainage, positive culture, local inflammatory signs with wound opening, or clinician diagnosis), (ii) deep incisional SSI (purulent drainage, wound dehiscence with fever/pain, abscess, or clinician diagnosis), and (iii) organ/space SSI (pelvic or intra-abdominal involvement with purulent drainage, positive culture, abscess, or clinician diagnosis; i.e., this category included post-CS endometritis) [24,25]. SSIs were further classified as occurring during hospital stay or in the post-discharge period, the latter identified through review of medical records up to one year postpartum for any clinical, diagnostic, or therapeutic evidence of infection consistent with the definitions. Two of the authors (AČJ and SJ) reviewed medical records and classified cases. Post-CS urinary tract infections were excluded from the analysis.

### 2.2. Surgical Site Infection Prevention Bundle and Surgical Technique

Preoperative skin preparation was performed as per hospital protocol by optional hair clipping and skin disinfection with 70% isopropyl alcohol supplemented with 2% chlorhexidine solution applied three times for a typical contact time of 60 s, which are used for CS in IGH. Vaginal cleansing was not routinely performed pre-CS. As for the surgical technique, Pfannenstiel or modified Joel-Cohen laparotomy is used. The subcutaneous tissue is dissected, the fascia is incised, and the peritoneal cavity is bluntly entered. Uterus is entered through a transverse lower uterine incision, membranes are ruptured, and myometrial incision is bluntly extended. After the fetus is extracted, the uterine cavity is inspected manually, and the cervix is dilated if necessary. The uterus is repaired with a single layer continuous suture outside the abdominal cavity. The abdominal cavity is cleaned without irrigation, and hemostasis is secured with hemostatic sutures if required. The peritoneum is left unsutured, rectus muscles are approximated optionally, the fascia is closed in one continuous layer, the subcutaneous tissue is approximated, and the skin is closed intradermally. Postoperatively, patients are encouraged to take shower on day 1, the wound is left uncovered from day 3 after which general hygiene measures are advised. Hospital discharge usually occurs on day 5. Before discharge, patients were instructed to carefully monitor their surgical wound and to ensure that any wound-related or other complications occurring within the first month were evaluated at our institution rather than by another physician.

### 2.3. Statistical Analysis

Descriptive statistics were used to summarize sample characteristics. SSI incidence was calculated and stratified by year. Crude odds ratios (ORs) and 95% confidence intervals (CIs) were estimated using univariable logistic regression. Predictors with a *p*-value less than 0.25 were subsequently included in the multivariable analysis with a forward stepwise selection method. Only predictors that significantly improved the model were retained in the final model. Results are reported as crude and adjusted ORs with 95% CI. A *p*-value less than 0.05 was considered statistically significant. A post hoc sample size analysis indicated that 216 patients would be required to detect a reduction from 8% to 1% with an alpha of 0.05 and a study power of 0.70.

## 3. Results

During the study period, a total of 1055 deliveries were recorded, including 517 before and 538 after the implementation of universal CS antimicrobial prophylaxis. The proportion of CS remained similar in both periods (19.1% vs. 20.3%, *p* = 0.650). Maternal characteristics, including mean age (31 vs. 30 years), parity, smoking status, BMI, and maternal weight at delivery, did not differ significantly between groups (Table 1).

Among women undergoing cesarean delivery, the proportion receiving antimicrobial prophylaxis increased markedly from 58.6% before to 89.0% after implementation (*p* < 0.001). Other perioperative factors such as emergency operations, type of anesthesia, and duration of surgery were comparable across the two periods.

The incidence of SSI was moderate overall (7.2%). It decreased in the second period of the study, after the implementation of universal antimicrobial prophylaxis, but the difference did not reach statistical significance (8.1% vs. 6.4%, *p* = 0.644). Most SSIs were superficial incisional infections; no deep infections were observed in either period. Most SSIs (87%) were diagnosed after hospital discharge, typically during the second postoperative week (median 11 days after CS). In the vast majority of cases (73%), no microbiological culture results were available, and all patients with SSI received systemic antimicrobial therapy.

In univariate logistic regression analyses, several parameters, such as antimicrobial prophylaxis, PROM duration, maternal weight, emergency surgery, duration of surgery and BMI showed borderline associations with SSI (all *p* < 0.25), while other factors such as smoking status and type of anesthesia were far from significance cut-off (Table 2).

After inclusion of all eligible variables in the multivariable logistic regression and forward stepwise selection, three factors remained independently associated with SSI. Antimicrobial prophylaxis was associated with a significant reduction in odds of SSI (adjusted OR 0.11; 0.02–0.58; *p* = 0.009). Longer PROM duration increased the odds of SSI (adjusted OR 1.06 per hour; 1.01–1.10; *p* = 0.009). Higher maternal weight was also significantly associated with increased odds of SSI (adjusted OR 1.04 per kg; 1.01–1.08; *p* = 0.014). No other factors remained in the final adjusted model.

## 4. Discussion

We report a moderate post-cesarean SSI rate of 7.2% in a community secondary care regional teaching hospital with a non-significant decrease in infection after the introduction of universal antimicrobial prophylaxis. Nevertheless, antibiotic prophylaxis significantly reduced the odds of SSI after cesarean delivery with PROM duration and maternal weight (but not BMI) as two additional predictors for SSI in the multivariable analysis. Systematic 30-day surveillance was essential for correct estimation of SSI burden as most infections were detected post-discharge (87%). Majority of infections were superficial incisional SSI while the reminder comprised endometritis classified under the category organ/space SSI. Our findings support guideline-concordant universal prophylaxis for cesarean delivery.

Adherence to antimicrobial prophylaxis guidelines varies across clinical settings and is influenced by institutional policies, as well as individual knowledge, attitudes, and beliefs [26]. With this in mind, the incidence of SSI is an important quality indicator in surgery and obstetrics, guiding evidence-based strategies for improvement. However, in the absence of routine surveillance, the true burden of SSI is often underestimated. The ECDC recently reported very low incidence of post-cesarean SSI with only 1.3% based on reported data from over 100,000 cesarean deliveries [12]. A recent Slovenian study including data from four Slovenian hospitals reported pooled in-hospital SSIs incidence of 1.9%, with 2/4 hospitals reported 0% incidence [13]. Importantly, only two of the four participating hospitals included in this audit reported data on SSI occurring after hospital discharge which most probably led to an underestimation of actual post-SC SSI rates. This may partly explain the prevailing perception that post-cesarean SSI are rare in Slovenia and why antimicrobial prophylaxis has traditionally been reserved for emergency procedures.

To address this gap, we introduced a systematic 30-day surveillance of post-cesarean SSI in early 2023 at our institution, which revealed an incidence close to 10%, considerably higher than expected. These findings prompted an institutional action plan, including a review of international guidelines, resulting in the adoption of universal antimicrobial prophylaxis. In 2024, adherence among 15 surgeons reached approximately 90%. After refining case definitions and excluding confirmed urinary tract infections, SSI incidence was 8.1% (95% CI, 3.6–15.3%) before the change and 6.4% (95% CI, 2.6–12.8%) after implementation. The limited sample size and incomplete adherence to prophylaxis likely reflects why this reduction did not reach statistical significance.

Our results should be interpreted cautiously, as retrospective studies often have significant variability and biases that can greatly influence reported SSI incidence. Published data are highly heterogeneous due to differences in clinical settings, study designs, case definitions, and surveillance methods [27]. Nevertheless, there is a general rule, that studies using standardized 30-day post-discharge follow-up tend to show rates comparable to ours [9,28,29,30,31], while those relying solely on inpatient data substantially underestimate the true burden as up 70% of SSI could be detected post-discharge in different studies, (87% in our case) [32,33,34]. These findings underscore the need for comprehensive surveillance systems and reveal significant potential for enhancing infection prevention strategies. Nevertheless, certain clinical settings report markedly lower incidence rates (<2%) than those observed in our study, suggesting that unmeasured local factors—such as surgical technique, patient characteristics, or follow-up methods—may play a critical role. Our results further indicate that achieving a reduction is not only possible but also necessary [12,32,35]. Finaly, a Norwegian study reported data from a setting comparable to Slovenia, where some hospitals used universal prophylaxis and others restricted it to high-risk CSs [30]. They found that hospitals implementing universal prophylaxis achieved higher compliance and significantly lower rates of superficial SSI in planned CSs. In contrast, no significant difference was observed in deep SSI rates or among emergency CSs, likely due to the already high prophylaxis use in these cases.

In a multivariable analysis, three factors were independently associated with the risk of SSI: antimicrobial prophylaxis, PROM duration, and maternal weight. The protective effect of antimicrobial prophylaxis in our study supports international recommendations advocating its routine use for all cesarean deliveries [21,22]. The adjusted odds ratio for antibiotic prophylaxis is even higher than in most studies, although with a wide confidence interval (0.11, 95% CI 0.02–0.58). We believe this may be explained by confounding by indication, as higher prophylaxis rates were achieved in high-risk cases before implementation, particularly in emergency operations. Precisely for this reason, in clinical settings where prophylaxis is used primarily for emergency procedures, the protective effect may initially appear more pronounced in elective operations, as reported in the aforementioned study [30]. Prolonged PROM was a strong predictor of SSI in our cohort, consistent with natural pathogenesis of infections, particularly endometritis, which was in our case represented in organ/space SSIs, representing 40% of all recorded SSIs. Best management of PROM is still somehow controversial and depends on maturity and clinical setting [36,37,38]. In our secondary care hospital, we administer antibiotic prophylaxis with cefazolin after 12 h from PROM at term and wait 48 h for induction. Women with preterm PROM at less than completed 34 weeks of gestation are transferred to a tertiary perinatal center. Finally, higher maternal weight was independently associated with SSI, consistent with previous reports linking obesity to an increased risk of surgical wound complications [39]. This association may be influenced by various patient- or procedure-related factors. However, it may also reflect altered pharmacokinetics of prophylactic antibiotics, particularly the possibility that an adequate prophylactic concentration may not be achieved in the surgical field in patients with greater mass [40,41,42]. This interpretation is further supported by the finding that BMI was not an independent risk factor, even though there is a strong collinearity between weight and BMI. In our analysis, absolute weight served as a more direct proxy for the volume of distribution of the prophylactic antibiotic reflected also by more narrow confidence interval in univariate analysis. Among our SSI subgroup of patients, 8/15 had ≥90 kg at delivery. More data is needed to determine a safe cut-off weight value and possible higher dose of cefazolin (3 g or higher) [43]. Additionally, other strategies were used to decrease the SSI in obese patients, such as prolonging antibiotic prophylaxis to 48 h [44] or extending the spectrum of prophylaxis with additional antibiotics such as metronidazole or azithromycin [43,44], both with favorable outcomes. Other factors, including emergency surgery, anesthesia type, smoking status, and surgery duration, were not significantly associated with SSI in our cohort. This may reflect the relatively uniform surgical practices and perioperative care at our institution, as well as the limited sample size, which may have reduced statistical power for detecting smaller effects.

These findings highlight the importance of universal antibiotic prophylaxis for CS. Moreover, with the identification of two additional independent risk factors, they support a reevaluation of optimal strategies for patients with PROM locally and potential adjustments of prophylactic antibiotic use, particularly for obese patients with either strategy. Our results also underscore the need for ongoing surveillance of post-cesarean SSIs, ideally at a national level. The National Perinatal Information System, established in Slovenia in 1987, currently records over 140 variables in a computerized database, including delivery type. Incorporating additional data on surgical prophylaxis practices and SSI occurrence would further strengthen efforts to promote safe CSs. With the current expertise and knowledge, a realistic goal is to further reduce post-cesarean SSI from the current approximate 6% to below 3% locally and possibly nationally.

Our study has several limitations. First, it was a relatively small, single-center study with potential biases inherent to the study, which may limit the generalizability of the findings. Second, not all surgeons fully adhered to the implemented protocol of universal prophylaxis. This lack of full compliance may have contributed to the absence of statistical significance and highlights the challenges of effective change management. Third, we lacked data on the exact timing of antibiotic administration prior to delivery, which is critical in SSI prevention. We acknowledge that adherence to administration within 60 min before incision, as per protocol, may have been better in cases of elective caesarean section. Fourth, SSIs were identified through medical records rather than direct follow-up (e.g., telephone contact) with women within 30 days after CS. This approach may underestimate the true incidence of SSI, as women with mild superficial wound infections may not have sought medical attention. Nevertheless, patients were specifically instructed regarding wound complication evaluations, which may have substantially reduced risk of under-ascertainment bias. Finally, as this is the first report of its kind in Slovenia, the results should be interpreted with caution. Nevertheless, they provide valuable evidence supporting the need for further investigation and potential changes in clinical practice.

## 5. Conclusions

Antibiotic prophylaxis was independently associated with a reduced risk of SSI following SC; however, the absolute infection rate did not decline significantly and remained moderate after implementation. Longer PROM-to-delivery interval and higher maternal weight were also identified as independent risk factors for SSI. These findings support the adoption of guideline-recommended universal prophylaxis and underscore the need to optimize management strategies for high-risk groups, particularly women with PROM or obesity. Continued surveillance at both the hospital and national levels is essential to accurately track SSI trends and further enhance the safety of CS.

## Figures and Tables

**Table 1:** Demographic and Clinical Characteristics Before and After Implementation of Universal Antimicrobial Prophylaxis.

	Before (2023)	After (2024)	*p*-Value
	No. (%)	No. (%)	
**All Deliveries**			
No. deliveries	517 (100)	538 (100)	/
Maternal age, mean (range)	31 (18–47)	30 (17–49)	0.336
Nulliparous	257 (49.7)	268 (49.8)	0.973
Non-smokers	460 (89.0)	467 (86.8)	0.280
**Cesarean Sections (CSs)**			
No. CS (% of all)	99 (19.1)	109 (20.3)	0.650
Maternal age, mean (range)	32 (31–33)	32 (31–33)	0.527
Nulliparous	52 (52.5)	57 (52.3)	0.974
Maternal weight at delivery, mean (range; kg)	86 (63–140)	89 (51–168)	0.236
Maternal body mass index at delivery, mean (range)	31 (23–51)	31 (18–54)	0.591
Duration of CS, mean (range; min)	49 (30–95)	51 (30–165)	0.411
No. surgeons	15	15	/
Emergency operation	60 (60.6)	54 (49.5)	0.110
General anesthesia	45 (45.5)	39 (35.8)	0.156
Regional anesthesia	54 (54.5)	70 (65.4)	0.156
Antimicrobial prophylaxis	58 (58.6)	97 (89.0)	<0.001
PROM * duration, mean (range; h)	6.3 (0–81)	3.8 (0–56)	0.124
**Surgical Site Infections (SSI)**			
No. SSI *	8 (8.1; 3.6–15.3)	7 (6.4; 2.6–12.8%)	0.644
Days after surgery	10 (2–18)	12 (4–26)	0.531
Superficial incisional SSI	5 (62.5)	4 (57.1)	/
Deep incisional SSI	0 (0)	0 (0)	/
Organ/space SSI	3 (37.5)	3 (42.9)	/
Pre-discharge diagnosis	2 (25.0)	0 (0.0)	/
Post-discharge diagnosis	6 (75.0)	7 (100)	/
Culture results available	3 (37.5)	1 (14.3)	/
Antibiotic therapy	8 (100.0)	7 (100.0)	/

* PROM prelabor rupture of membranes.

**Table 2:** Crude and Adjusted Odds Ratios for Factors Associated with Surgical Site Infection after Cesarean Section.

Risk Factors	Odds Ratio (95% CI)			
	Crude	*p*-Value	Adjusted *	*p*-Value
Antimicrobial prophylaxis	0.35 (0.12–1.04)	0.059	0.11 (0.02–0.58)	0.009
PROM * duration	1.02 (0.99–0.05)	0.213	1.06 (1.01–1.10)	0.009
Maternal weight at delivery	1.02 (1–1.05)	0.067	1.04 (1.01–1.08)	0.014
Emergency operation	0.53 (0.18–1.53)	0.238	/	/
Maternal body mass index at delivery	1.05 (0.97–1.14)	0.226	/	/
Duration of surgery	1.02 (0.99–1.05)	0.153	/	/
General anesthesia	1.32 (0.46–3.78)	0.608	/	/
Smoking	0.93 (0.75–1.16)	0.539	/	/

* Adjusted odds ratios were derived from a multivariable logistic regression using forward stepwise selection method; only variables that remained significant were retained in the final model. PROM prelabor rupture of membranes before cesarean section; CI confidence interval.

## Data Availability

The data are not publicly available due to ethical restrictions.

## Abbreviations

The following abbreviations are used in this manuscript:

CS	Cesarean section
SSI	Surgical site infection
NPIS	The National Perinatal Information System
IGH	Izola General Hospital
